# ATP-Responsive Bimetallic Metal–Organic Frameworks Amplify Oxidative Stress in the Tumor Microenvironment for Synergistic Chemo-Immunotherapy

**DOI:** 10.3390/jfb17040199

**Published:** 2026-04-19

**Authors:** You Li, Wenxin Zhang, Zitao Xu, Shixin Ma, Yufei Xiong, Li Yu, Huiling Gao, Yang Shu, Teng Fei

**Affiliations:** 1 Key Laboratory of Bioresource Research and Development of Liaoning Province, College of Life and Health Sciences, Northeastern University, Shenyang 110819, China; nanjiliyou@163.com (Y.L.); zitaox1025@163.com (Z.X.); mashixin0902@163.com (S.M.); xiong030616@outlook.com (Y.X.); yu_li2017@163.com (L.Y.); gaohuiling@mail.neu.edu.cn (H.G.); 2 National Frontiers Science Center for Industrial Intelligence and Systems Optimization, Northeastern University, Shenyang 110819, China; 3 Key Laboratory of Data Analytics and Optimization for Smart Industry (Northeastern University), Ministry of Education, Shenyang 110819, China; 4 Department of Chemistry, College of Sciences, Northeastern University, Shenyang 110819, China; 20242264@cmu.edu.cn

**Keywords:** bimetallic MOFs, chemodynamic therapy, oxidative stress, cGAS, STING, tumor microenvironment, immunotherapy, ion interference

## Abstract

Metal ion-based chemo-immunotherapy is often limited by rigid intracellular metal homeostasis, insufficient reactive oxygen species (ROS) accumulation, and an immunosuppressive tumor microenvironment (TME). To overcome these limitations, we engineered an ATP-responsive, core–shell bimetallic nanoreactor (Cu/ZIF@PDA, termed CZP) featuring a precisely controlled ~25 nm biomimetic polydopamine (PDA) coating. Triggered by elevated tumoral ATP levels, CZP undergoes coordination-induced disassembly and promotes oxidative stress amplification. Specifically, the PDA shell acts as a superoxide dismutase (SOD) mimetic to continuously supply H_2_O_2_, fueling Cu^2+^-mediated Fenton-like reactions to unleash highly toxic hydroxyl radicals (•OH) while aggressively depleting the intracellular glutathione (GSH) pool. This irreversible oxidative damage, coupled with Zn^2+^-induced mitochondrial dysfunction, triggers profound mitochondrial DNA (mtDNA) leakage. Crucially, this cytosolic DNA robustly activates the cGAS-STING signaling axis, driving a massive surge in immunogenic cell death (ICD) and significantly promoting dendritic cell (DC) maturation. Furthermore, CZP markedly inhibited primary tumor growth in vivo and showed protection in a tumor re-challenge model, accompanied by enhanced dendritic cell maturation. These findings support the potential of this ATP-responsive bimetallic nanoplatform to promote antitumor immune activation.

## 1. Introduction

Cancer remains a major threat to human health worldwide [1,2]. Traditional clinical treatments, such as chemotherapy and radiotherapy, are widely used. However, they often cause severe side effects to normal tissues and easily lead to drug resistance [3,4]. In recent years, tumor immunotherapy has developed rapidly. For example, immune checkpoint blockade (ICB) therapy has shown great success in clinical trials [5,6,7]. It works by activating the patient’s own immune system to attack cancer cells. Unfortunately, only a small number of patients respond well to ICB therapy. Most solid tumors fail to respond because they are “cold” tumors [8,9]. These tumors lack the infiltration of immune cells, especially cytotoxic T lymphocytes. In addition, the tumor microenvironment (TME) is highly immunosuppressive. The TME features dense physical barriers, low oxygen levels, and abnormal metabolism [10,11,12]. This complex environment actively stops immune cells from entering and functioning. Therefore, finding a way to reverse this immunosuppressive TME and turn “cold” tumors into “hot” tumors is a critical challenge [13,14,15].

To transform “cold” tumors into “hot” ones, it is essential to effectively kill tumor cells and alert the immune system. Recently, metal-based nanomedicines have shown great promise for this task [16,17]. They mainly rely on chemodynamic therapy (CDT) [18,19,20]. In CDT, metal ions act as catalysts to produce highly toxic reactive oxygen species (ROS). These ROS can directly destroy tumor cells and stimulate immune responses [21,22,23]. However, the complex TME greatly limits the actual effect of traditional CDT. First, CDT reactions need hydrogen peroxide (H_2_O_2_) as fuel. But the natural H_2_O_2_ level in the TME is very low [24]. Second, tumor cells produce large amounts of GSH. GSH is a strong antioxidant that quickly clears out the generated ROS [25,26]. More importantly, simply producing surface-level ROS is usually not enough to activate deep immune pathways. To achieve strong and long-lasting anti-tumor immunity, activating the cyclic GMP-AMP synthase (cGAS)-stimulator of interferon genes (STING) pathway is highly necessary [27,28,29,30,31]. This pathway senses severe cell damage and acts as a vital bridge to wake up the whole immune system. Therefore, there is an urgent need for a smart platform that can overcome these TME barriers, massively amplify ROS, and deeply activate the cGAS-STING pathway [32,33].

To meet these clinical needs, we developed an adenosine triphosphate (ATP)-responsive bimetallic metal–organic framework (MOF) nanoplatform (Cu/ZIF@PDA, CZP) with a biomimetic polydopamine (PDA) shell, which integrates Cu/Zn dual metal ion interference and PDA enzyme-mimetic activity for synergistic chemo-immunotherapy. Specifically, this ATP-responsive bimetallic MOF enables tumor-specific intracellular disassembly and cascade catalysis. Following disassembly, the Cu/PDA components synergistically amplify ROS generation by self-supplying H_2_O_2_ to bypass endogenous limitations and aggressively depleting intracellular GSH. This synergistic oxidative stress, coupled with zinc-induced mitochondrial dysfunction, triggers severe mtDNA leakage to robustly activate the cGAS-STING pathway. Ultimately, this targeted therapy promotes the maturation of dendritic cells (DCs) and establishes long-term immunological memory to prevent tumor recurrence. In summary, this smart bimetallic platform provides a highly effective and streamlined strategy to remodel the TME for advanced cancer immunotherapy.

## 2. Materials and Methods

### 2.1. Materials and Reagents

Zinc nitrate hexahydrate (Zn(NO_3_)_2_·6H_2_O, #Z118841), 2-methylimidazole (2-MIM, #M104839), copper chloride dihydrate (CuCl_2_·2H_2_O, #C111683), dopamine hydrochloride (DA, #D590946), and glutathione (GSH, #G105427) were purchased from Aladdin Biochemical Technology Co., Ltd. (Shanghai, China). ATP (#797189), 5,5′-dithiobis-(2-nitrobenzoic acid) (DTNB, #D8130), methylene blue (MB, #457250), and Cell Counting Kit-8 (CCK-8, #96992) were obtained from Sigma-Aldrich (St. Louis, MO, USA). All other chemicals were of analytical grade and used as received.

For Western blot analysis, primary antibodies against TBK1 (#CPA5544), p-TBK1 (#CPA5532), IRF3 (#CPA4965), p-IRF3 (#CPA6471), and p-STING (#CPA6699) were purchased from Cohesion Biosciences (Suzhou, China). The primary antibody against GAPDH (#sc-25778) was obtained from Santa Cruz Biotechnology (Dallas, TX, USA). Goat anti-Rabbit IgG (#31460) and Goat anti-Mouse IgG (#A-11001) were purchased from Thermo Fisher Scientific (Waltham, MA, USA). For flow cytometry, fluorochrome-conjugated antibodies against CD80-Cy5.5 (#15-0801-82), CD86-FITC (#48-0862-82), and CD11c-BV605 (#53-0114-82) were acquired from BD Biosciences (San Jose, CA, USA).

Mouse 4T1 cells were obtained from ATCC (Manassas, VA, USA; #CRL-2539). Recombinant murine GM-CSF (#HY-P7069), IL-4 (#HY-P7080), FAM (#HY-W021817), WST-1 (#HY-136976), Calcein-AM (#HY-D0041), and DCFH-DA (#HY-D0940) were purchased from MedChemExpress (Monmouth Junction, NJ, USA). The Annexin V-FITC/PI apoptosis detection kit (#C1062S) was purchased from Beyotime Biotechnology (Shanghai, China).

### 2.2. Synthesis of Cu/ZIF@PDA Nanoparticles

The Cu-doped ZIF-8 (hereafter referred to as CZ) nanoparticles were synthesized via a one-pot self-assembly strategy. In a typical procedure, 1.54 g of 2-MIM was dissolved in 8 mL of deionized water. Separately, 60 mg of Zn(NO_3_)_2_·6H_2_O and 17.16 mg of CuCl_2_·2H_2_O were each dissolved in 1 mL of water. The metal salt solutions were mixed and then added dropwise into the ligand solution under vigorous stirring. After reacting for 5 min, the precipitates were collected by centrifugation (8500 rpm, 10 min), washed with methanol, and vacuum dried. To construct the core–shell PDA-coated CZ (referred to as CZP in figures), the as-synthesized CZ nanoparticles were redispersed in 20 mL of Tris-HCl buffer (50 mM, pH 8.5) containing 10 mg of dopamine. The mixture was stirred for 12 min to initiate the in situ self-polymerization of dopamine. The final products were harvested by centrifugation (12,000 rpm, 12 min) and washed extensively with water.

### 2.3. Nanoparticle Characterization

Morphology and microstructure were visualized using scanning electron microscopy (SEM, Hitachi SU8010, Hitachi High-Tech Corporation, Tokyo, Japan, operating at an accelerating voltage of 5.0 kV) and transmission electron microscopy (TEM, Tecnai G20, FEI Company, Hillsboro, OR, USA, operating at 200 kV). Crystal structures were analyzed by X-ray diffraction (XRD) using a Bruker D8 Advance diffractometer (Bruker AXS, Karlsruhe, Germany) with Cu Kα radiation (λ = 1.5406 Å) over a 2θ range of 5° to 50°. Chemical compositions and valence states were determined using Fourier transform infrared spectroscopy (FT-IR, Nicolet-6700, Thermo Electron Corporation, Madison, WI, USA) and X-ray photoelectron spectroscopy (XPS, AXIS-SUPRA, Kratos Analytical Ltd, Manchester, UK). Hydrodynamic size and zeta potential distributions were measured on a Zetasizer Nano ZS90 (Malvern Instruments Ltd., Malvern, Worcestershire, United Kingdom).

The Cu content in the CZ sample was quantified by inductively coupled plasma mass spectrometry (ICP-MS, Agilent 8900, Agilent Technologies, Santa Clara, CA, USA). Briefly, 37.00 mg of CZ was completely digested in trace-metal-grade nitric acid and diluted with deionized water to an appropriate concentration range before analysis. Cu concentration was determined using an external calibration curve, and the Cu content in the original sample was calculated after correction for the dilution factor. The results were expressed as μg/mg sample and wt%.

### 2.4. ATP-Triggered Disassembly

To evaluate ATP responsiveness, 5 mg of CZP nanoparticles were incubated with 10 mL of FAM solution (50 µg/mL) for 1 h under magnetic stirring to ensure binding. After removing unbound FAM through centrifugation and washing twice with PBS, the FAM-labeled CZP nanoparticles were resuspended in PBS (1 mg/mL) and incubated with varying concentrations of ATP (0, 0.5, and 2 mM) at 37 °C for 2.5 h. The fluorescence intensity was measured at 517 nm to quantify the cumulative release of FAM, reflecting the structural degradation of the MOF.

### 2.5. Evaluation of Cascade Catalytic Performance

#### 2.5.1. GSH Depletion

The GSH-consuming capability was assessed using Ellman’s reagent (DTNB). CZP suspensions were incubated with GSH (2 mM) at 37 °C. The residual GSH was quantified by measuring the absorbance of the reaction product (TNB) at 412 nm.

#### 2.5.2. SOD-Mimicking Activity

SOD-like activity was evaluated using a WST-1 assay kit. The inhibition rate of WST-1 formazan formation was calculated to determine the ability of the nanoparticles to scavenge superoxide anions (O_2_^•−^).

#### 2.5.3. •OH Generation

Hydroxyl radical production was monitored using the methylene blue (MB) degradation assay. CZP was incubated with GSH (10 mM) and H_2_O_2_ (5 mM). The decrease in absorbance at 665 nm was recorded at predetermined intervals to evaluate the Fenton-like catalytic efficiency.

### 2.6. In Vitro Cytotoxicity and Intracellular ROS Detection

The murine breast cancer cell line 4T1 was cultured and seeded into flat-bottom 96-well plates at a density of 1 × 10^4^ cells per well. 4T1 cells were cultured in RPMI-1640 supplemented with 10% FBS and 1% penicillin-streptomycin at 37 °C in 5% CO_2_. Cells were treated with ZIF-8, CZ, or CZP at various concentrations for 24 h. Cell viability was determined using the CCK-8 assay. For live/dead visualization, cells treated for 16 h were co-stained with Calcein-AM and Propidium iodide (PI), and imaged using confocal laser scanning microscopy (CLSM, Olympus FV1200, Olympus Corporation, Tokyo, Japan). Apoptosis rates were quantified via flow cytometry (BD LSRFortessa, BD Biosciences, San Jose, CA, USA) using an Annexin V-FITC/PI apoptosis detection kit. Intracellular ROS levels were assessed using the DCFH-DA probe (10 μM). After treatment, the fluorescence of oxidized DCF was analyzed by both CLSM and flow cytometry.

### 2.7. Western Blot Analysis

4T1 cells (1 × 10^6^ cells per dish) were treated with PBS, ZIF-8, CZ, or CZP for 24 h. Total proteins were extracted using 100 µL of RIPA lysis buffer supplemented with protease/phosphatase inhibitors and quantified via the BCA method. Equal amounts of protein were separated by SDS-PAGE, transferred to PVDF membranes, and blocked. The membranes were incubated overnight at 4 °C with primary antibodies against p-STING, TBK1, p-TBK1, IRF3, p-IRF3, and GAPDH, followed by HRP-conjugated secondary antibodies. Protein bands were visualized using an enhanced chemiluminescence (ECL) system (Tanon 5200, Shanghai Tanon Life Science Co., Ltd., Shanghai, China).

### 2.8. Assessment of Dendritic Cell (DC) Maturation

Bone marrow-derived dendritic cells (BMDCs) were isolated from BALB/c mice and differentiated with GM-CSF (20 ng/mL) and IL-4 (10 ng/mL) for 7 days. To evaluate ICD-mediated DC maturation, immature DCs were co-cultured with 4T1 cells in the presence of ZIF-8, CZ, or CZP for 24 h. The DCs were then stained with fluorochrome-conjugated antibodies against CD11c (BV605), CD80 (Cy5.5), and CD86 (FITC). The proportion of mature DCs (CD11c^+^CD80^+^CD86^+^) was analyzed by flow cytometry.

### 2.9. In Vivo Antitumor Therapy and Re-Challenge

All animal procedures were performed according to the Guidelines for the Care and Use of Laboratory Animals and were approved by the Biological and Medical Ethics Committee of Northeastern University (Shenyang, China, NEU-EC-2021A020S). A subcutaneous tumor model was established by injecting 5 × 10^5^ 4T1 cells into the right flank of immunocompetent BALB/c mice. When tumor volumes reached approximately 50–80 mm^3^, mice were randomized into four groups (*n* = 6): PBS, ZIF-8, CZ, and CZP. Treatments were administered intravenously on days 0, 2, and 4 (equivalent Cu dose: 4 mg/kg). Tumor volume (V = 0.5 × length × width^2^) and body weight were monitored every other day. On day 8, tumors were harvested for weighing and photographing. For the re-challenge experiment, CZP was selected for follow-up evaluation based on the primary efficacy screening. Therefore, primary tumors in the PBS and CZP groups were surgically resected on day 8. On day 15, surviving mice were re-inoculated with 5 × 10^5^ 4T1 cells on the contralateral (left) flank. The growth of secondary tumors was monitored for a period of 7 days to evaluate whether CZP treatment was associated with measurable re-challenge protection.

### 2.10. In Vivo Immune Analysis

To analyze the tumor immune microenvironment, tumors were harvested at the end of treatment and digested into single-cell suspensions. The cell suspensions were stained with a panel of fluorochrome-conjugated antibodies: CD11c (BV605), CD80 (Cy5.5), and CD86 (FITC). Data acquisition and analysis were performed using a flow cytometer (BD LSRFortessa, BD Biosciences, San Jose, CA, USA) and FlowJo software v10.8.1 (FlowJo LLC, Ashland, OR, USA).

### 2.11. TUNEL Staining of Tumor Sections

Excised tumor tissues were fixed, paraffin-embedded, and sectioned. The sections were deparaffinized in xylene, rehydrated through a graded ethanol series, and washed with PBS. After permeabilization with Proteinase K working solution at 37 °C for 20 min, the sections were equilibrated with Equilibration Buffer for 10 min at room temperature. TUNEL staining was then performed using a CF488 TUNEL Apoptosis Detection Kit (Servicebio, Wuhan, China, #G1504) according to the manufacturer’s instructions. Briefly, sections were incubated with the TdT labeling mixture at 37 °C for 1 h in a dark humidified chamber, followed by DAPI counterstaining for 10 min. After mounting with anti-fade medium, fluorescence images were acquired using an upright fluorescence microscope (Eclipse C1, Nikon Corporation, Tokyo, Japan). TUNEL-positive apoptotic cells showed green fluorescence, and nuclei were stained blue.

### 2.12. Statistical Analysis

Data are presented as mean ± standard deviation (SD). Comparisons among multiple groups at a single time point were analyzed using one-way analysis of variance (ANOVA) followed by Tukey’s post hoc test. Comparisons between two independent groups were performed using an unpaired Student’s *t*-test. For longitudinal data, including tumor growth and body-weight curves, statistical analysis was performed using two-way repeated-measures ANOVA. *p* < 0.05 was considered statistically significant (* *p* < 0.05, ** *p* < 0.01, *** *p* < 0.001).

## 3. Results

### 3.1. Synthesis, Characterization of CZP Nanoparticles

**Figure 1:**
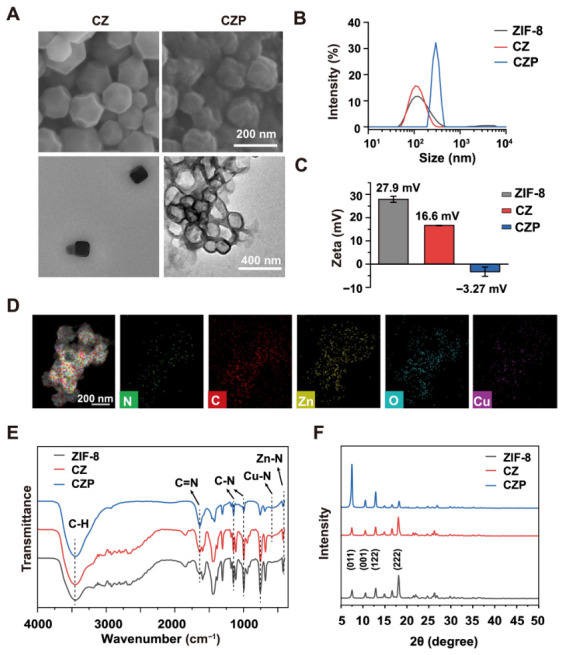
Synthesis and comprehensive characterization of the bimetallic CZP nanoplatform. (**A**) Representative scanning electron microscopy (SEM) and transmission electron microscopy (TEM) images of ZIF-8, CZ, and CZP nanoparticles, illustrating the uniform dodecahedral morphology and the distinct core–shell structure of CZP. (**B**) Hydrodynamic size distributions measured by dynamic light scattering (DLS), indicating the size expansion of CZP due to the swelling of the PDA shell in aqueous environment. (**C**) Zeta potential measurements of the nanoparticles, with the sharp charge reversal from positive to negative verifying the successful coating of the PDA shell. (**D**) Energy-dispersive X-ray spectroscopy (EDS) elemental mapping of a single CZP nanoparticle, demonstrating the uniform internal Cu doping and the presence of the external oxygen-rich PDA layer. The colored maps represent the spatial distributions of N, C, Zn, O, and Cu, respectively. (**E**) Fourier transform infrared (FTIR) spectra of the synthesized nanoparticles, revealing the emergence of the Cu-N peak as direct evidence of successful Cu^2+^ substitution within the framework. (**F**) X-ray diffraction (XRD) patterns of the synthesized powders, confirming the structural preservation of the highly ordered porous crystal framework after the doping and coating processes.

Scanning electron microscopy (SEM) and transmission electron microscopy (TEM) revealed the exact morphological features of these nanoparticles (Figure 1A). The CZ nanoparticles formed a uniform dodecahedral shape with an average size of approximately 165 nm. Subsequently, the CZ surface was coated with PDA through in situ polymerization to create the final CZP nanoparticles. TEM images showed that CZP possessed a core–shell structure with a PDA shell thickness of about 25 nm, and the overall particle size increased to approximately 196 nm.

The hydrodynamic size was then measured using dynamic light scattering (DLS). The DLS sizes of ZIF-8, CZ, and CZP were 105.7 nm, 122.4 nm, and 295.3 nm, respectively (Figure 1B). The hydrodynamic size of CZP was larger than that observed by TEM. Furthermore, the surface charge was evaluated using zeta potential measurements (Figure 1C). Pure ZIF-8 exhibited a positive charge of +27.9 mV, and the CZ nanoparticles showed a charge of +16.6 mV. After PDA coating, the zeta potential of CZP shifted from positive to negative (Figure 1C), consistent with successful surface modification.

Next, energy-dispersive X-ray spectroscopy (EDS) mapping was employed to visualize the spatial distribution of different elements within the CZP nanoparticles (Figure 1D). Strong signals for C, N, O, Zn, and Cu elements were clearly detected. The Cu signal was distributed throughout the particle core (Figure 1D). In contrast, the oxygen (O) signal was heavily concentrated on the outer edge of the nanoparticles. As the PDA molecule contains abundant oxygen-rich groups, this specific oxygen ring directly confirmed the presence of the external PDA shell.

Fourier transform infrared (FTIR) spectra of ZIF-8, CZ, and CZP are shown in Figure 1E. All samples maintained the typical C=N peak at 1590 cm^−1^, the C-N stretch peaks at 1145 and 991 cm^−1^, and the imidazole bending peak at 1309 cm^−1^. However, a critical difference in the metal-nitrogen bonds was detected. For pure ZIF-8, a strong Zn-N stretch peak appeared at 421 cm^−1^. In the CZ and CZP samples, the Zn-N peak became significantly weaker and a Cu-N peak appeared at 570 cm^−1^ (Figure 1E).

Finally, X-ray diffraction (XRD) confirmed the high structural stability of the synthesized materials (Figure 1F). The ZIF-8, CZ, and CZP powders all exhibited sharp diffraction peaks at 7.57, 10.67, 12.98, and 18.18 degrees. These angles were consistent with the (011), (001), (122), and (222) crystal planes of standard ZIF-8. The highly similar XRD patterns indicated that the original crystal structure remained completely intact. ICP-MS analysis further quantified the Cu incorporation in the CZ sample, showing a Cu content of 34.31 μg/mg (3.43 wt%), providing quantitative support for Cu incorporation into the framework (Table S1). These data collectively confirmed the successful preparation of CZP.

### 3.2. ATP-Responsive Disassembly and Cascade Catalytic Performance

**Figure 2:**
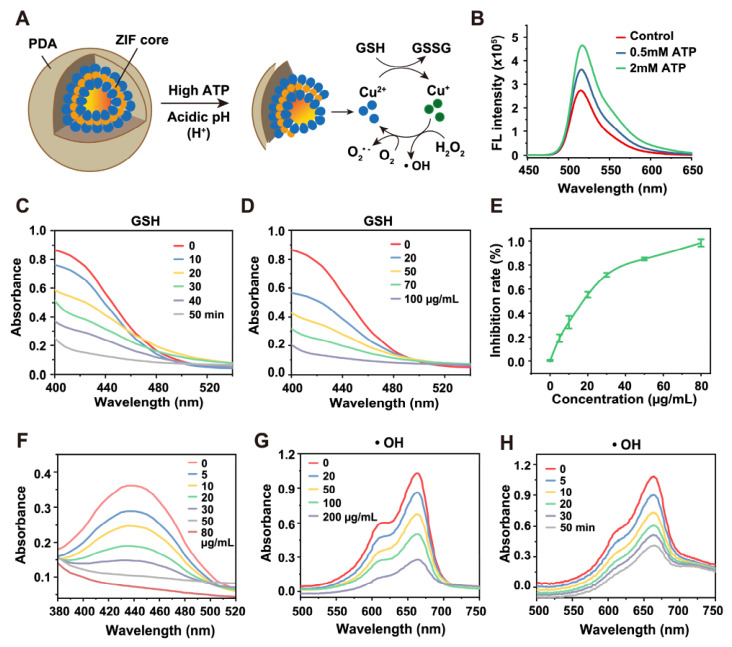
Catalytic properties and chemical reactivity of the nanoparticles. (**A**) Schematic illustration of the chemical reactions triggered by CZP, depicting the ATP-responsive disassembly and the subsequent cascade catalytic processes. (**B**) Fluorescence intensity of FAM-labeled CZP incubated at various concentrations, verifying the concentration-dependent disintegration of the framework. (**C**,**D**) Time-dependent (0–50 min) and concentration-dependent (0–200 μg/mL) GSH depletion by CZP, demonstrating the efficient antioxidant scavenging capability of the released active species. (**E**,**F**) Inhibition rate of WST-formazan formation and the corresponding SOD-like activity of CZP at varied concentrations (0–80 μg/mL), confirming the efficient catalytic conversion of superoxide anions into H_2_O_2_. (**G**,**H**) UV-vis absorption spectra of MB degradation induced by CZP across different concentrations for 30 min (**G**) and at different time points at 100 μg/mL (**H**). Data are presented as mean ± SD (*n* = 3).

ATP-responsive disassembly of CZP was next evaluated. As illustrated in Figure 2A, ATP was used as the trigger for framework disassembly. The fluorescence signal increased in an ATP concentration-dependent manner (Figure 2B), indicating ATP-responsive disassembly of the framework.

The catalytic activities of CZP were then evaluated. As shown in Figure 2E,F, CZP inhibited WST-formazan formation, indicating SOD-like activity. The Fenton-like activity was further assessed using an MB degradation assay, in which the blue color of MB rapidly faded in the presence of CZP (Figure 2G,H). In addition, the GSH level decreased after CZP treatment (Figure 2C,D). Together, these results showed that CZP exhibited SOD-like activity, promoted MB degradation, and depleted GSH.

### 3.3. In Vitro Anticancer Efficacy

**Figure 3:**
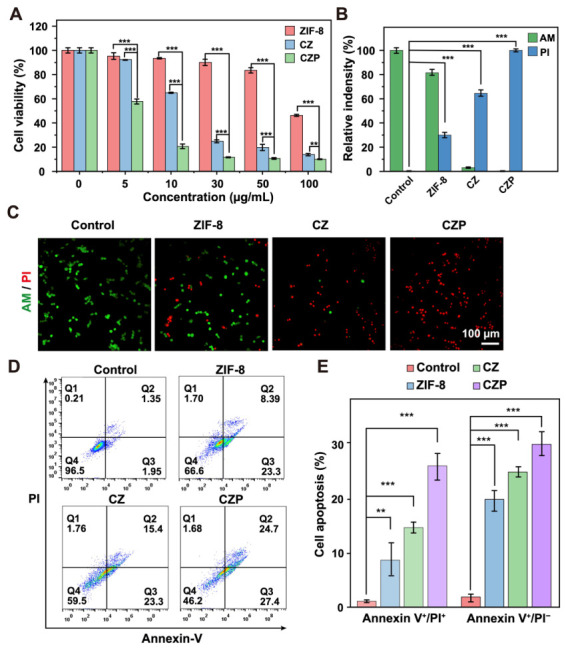
In vitro cytotoxicity and apoptosis-inducing capacity of the nanoparticles. (**A**) Cell viability of 4T1 cells treated with ZIF-8, CZ, and CZP at varied concentrations (0–100 μg/mL) for 24 h. (**B**,**C**) Representative fluorescence images of 4T1 cells co-stained with Calcein-AM (live cells) and PI (dead cells) following treatments with PBS, ZIF-8, CZ, and CZP (**C**), alongside the corresponding quantification of relative fluorescence intensities (**B**). Scale bar: 100 μm. (**D**,**E**) Flow cytometry analysis of apoptosis and necrosis in 4T1 cells after incubation with PBS, ZIF-8, CZ (100 μg/mL), and CZP (100 μg/mL) for 16 h (**D**) and the corresponding quantitative statistical analysis for early apoptotic cells (Annexin V^+^/PI^−^) and late apoptotic or necrotic cells (Annexin V^+^/PI^+^) (**E**). Pseudocolor indicates event density. Data are presented as mean ± SD (*n* = 3). ** *p* < 0.01, *** *p* < 0.001, assessed by one-way ANOVA followed by Tukey’s post hoc test.

The in vitro anticancer activity of CZP was then evaluated in 4T1 cells. First, a standard cell viability assay measured the general cytotoxicity. All copper-containing treatments showed a clear dose-dependent killing effect (Figure 3A). However, the CZP nanoparticles caused significantly higher cell death than the simple CZ nanoparticles at equivalent concentrations. To visually confirm this cell-killing effect, a live/dead co-staining method was used. PBS- and ZIF-8-treated cells showed predominantly green fluorescence (Figure 3C). In sharp contrast, the CZP treatment produced massive red fluorescence and caused a dramatic reduction in cell density. Flow cytometry further showed that CZP induced the highest levels of apoptotic and necrotic cell death among the tested groups (Figure 3D,E).

### 3.4. Intracellular ROS Generation, STING Pathway Activation, and Dendritic Cell Maturation In Vitro

**Figure 4:**
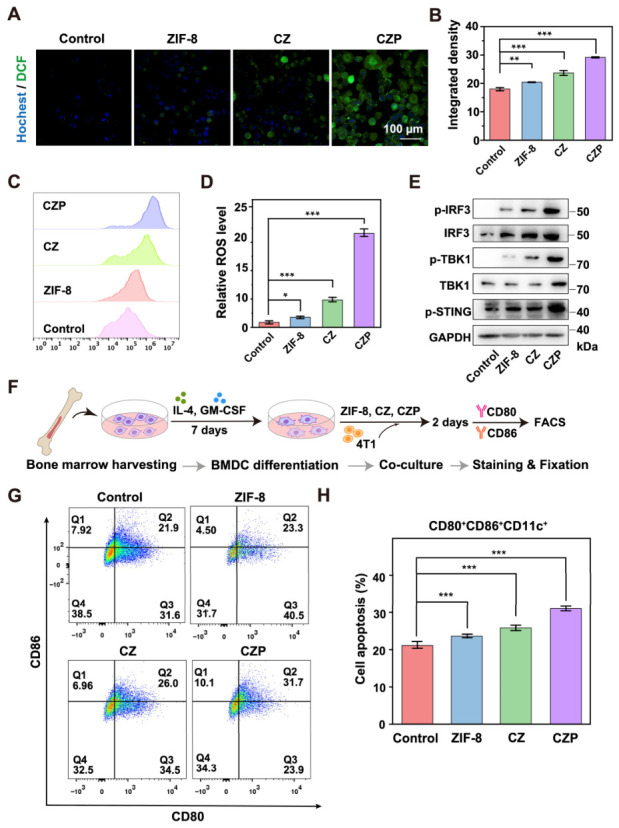
In vitro intracellular ROS generation, STING pathway activation, and dendritic cell maturation. (**A**,**B**) Representative confocal laser scanning microscopy (CLSM) images of intracellular ROS generation in 4T1 cells treated with PBS, ZIF-8, CZ, and CZP (**A**), alongside the corresponding quantitative analysis of relative fluorescence intensity (**B**). Scale bar: 100 μm. (**C**,**D**) Flow cytometry analysis of intracellular ROS generation in 4T1 cells following the aforementioned treatments (**C**) and the corresponding quantitative statistical analysis of relative mean fluorescence intensity (**D**). (**E**) Western blot analysis of STING pathway-related proteins (p-STING, p-TBK1, TBK1, p-IRF3, and IRF3) in 4T1 cells after different treatments. (**F**) Schematic illustration of the in vitro BMDC maturation assay. (**G**,**H**) Flow cytometry analysis of mature BMDCs (CD80^+^ CD86^+^ gated on CD11c^+^ cells) (**G**) and the corresponding quantitative statistical analysis (**H**) after the indicated treatments. Pseudocolor indicates event density. Data are presented as mean ± SD (*n* = 3). * *p* < 0.05, ** *p* < 0.01, *** *p* < 0.001 (one-way ANOVA followed by Tukey’s post hoc test).

Intracellular ROS generation was next examined. Under a confocal microscope, cells treated with PBS or pure ZIF-8 showed almost no green signal (Figure 4A,B). The CZP group showed the strongest fluorescence signal in both CLSM and flow cytometry analyses (Figure 4A–D), indicating increased intracellular ROS generation. Western blot analysis showed that CZP treatment increased the levels of p-STING, p-TBK1, and p-IRF3 compared with the control groups (Figure 4E). To examine the effect of tumor cells on dendritic cell (DC) maturation, a BMDC co-culture system was established (Figure 4F). Flow cytometry was utilized to detect specific maturation markers (CD80 and CD86) on the DC surface. The results demonstrated that the CZP treatment induced the highest rate of DC maturation among the tested groups (Figure 4G,H).

### 3.5. In Vivo Antitumor Efficacy and Biosafety of CZP

**Figure 5:**
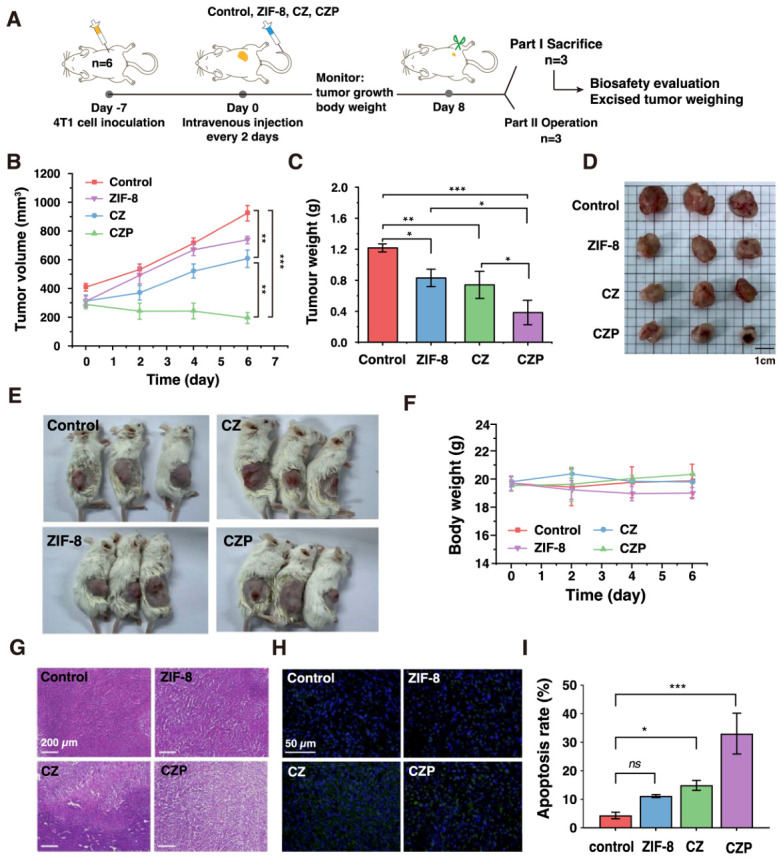
In vivo antitumor efficacy and biosafety of the nanoparticles. (**A**) Schematic illustration of the in vivo therapeutic protocol, depicting the treatment timeline and intravenous administration strategy for the 4T1 breast cancer model. (**B**) Tumor growth curves of 4T1 tumor-bearing mice receiving different treatments (PBS, ZIF-8, CZ, and CZP at a dose of 4 mg/kg) over time. Data are presented as mean ± SD (*n* = 6). (**C**,**D**) Average tumor weights (**C**) alongside the corresponding macroscopic photographs of the excised tumors (**D**) at the endpoint. Data are presented as mean ± SD (*n* = 3). (**E**) Representative photographs of tumor-bearing mice during the treatment period. (**F**) Body weight changes in mice during the treatment period. Data are presented as mean ± SD (*n* = 6). (**G**) Representative H&E staining images of the orthotopic tumor tissues excised after different treatments. Scale bar: 200 μm. (**H**) Representative fluorescence images of TUNEL-stained tumor sections from different treatment groups (blue: DAPI-stained nuclei; green: TUNEL-positive apoptotic cells). (**I**) Quantitative analysis of the relative TUNEL-positive area (apoptosis rate) across different groups. Data are presented as mean ± SD (*n* = 3). *ns*, not significant, * *p* < 0.05, ** *p* < 0.01, *** *p* < 0.001. For endpoint comparisons in (**C**,**I**), one-way ANOVA followed by Tukey’s post hoc test was used. For longitudinal data in (**B**), statistical analysis was performed using two-way repeated-measures ANOVA.

The in vivo antitumor efficacy of CZP was evaluated in a 4T1 tumor-bearing mouse model. Female BALB/c mice were subcutaneously injected with 4T1 cells. The mice were randomly divided into four groups. These groups received different treatments via intravenous injection: PBS, ZIF-8, CZ, and CZP (Figure 5A). Tumor growth was monitored throughout the treatment period until day 8. As expected, tumors in the PBS and ZIF-8 groups grew very rapidly (Figure 5B). The simple CZ treatment showed a moderate inhibitory effect. In sharp contrast, the CZP treatment almost completely suppressed the tumor growth. The endpoint tumor weights perfectly matched these volume measurements, and the excised tumors from the CZP group were significantly smaller than those from all other groups (Figure 5C,D). Notably, macroscopic observation of the mice provided direct visual evidence of this efficacy. Tumors in the CZP-treated group showed visible surface ulceration and central depression (Figure 5E).

Biosafety is an important consideration for in vivo evaluation. The body weights of all mice were recorded throughout the entire treatment period. No significant body weight loss was observed in any experimental group (Figure 5F). Furthermore, major organs (heart, liver, spleen, lung, and kidney) were collected for histological analysis (Figure S1B). Standard H&E staining showed no obvious tissue damage, necrosis, or severe inflammation in any tested organs.

Moreover, hematoxylin and eosin (H&E) staining of the tumor slices provided further pathological evidence. Compared to the dense cellular structure in the control groups, tumors from the CZP-treated mice exhibited severe structural looseness, extensive necrotic areas, and massive nuclear shrinkage (Figure 5G). To further evaluate therapy-induced cell death in vivo, TUNEL staining was performed on the tumor sections (Figure 5H,I). Consistent with the H&E staining results, tumors from the PBS and ZIF-8 groups exhibited minimal TUNEL-positive signals. The CZ treatment induced a moderate level of apoptosis. In contrast, the CZP-treated tumor tissues displayed significantly increased TUNEL-positive signals colocalized with DAPI-stained nuclei, indicating marked cellular apoptosis. These immunohistochemical findings are consistent with the macroscopic tumor regression observed following CZP treatment.

### 3.6. Re-Challenge Protection and Tumor Immune Activation Associated with CZP Treatment

**Figure 6:**
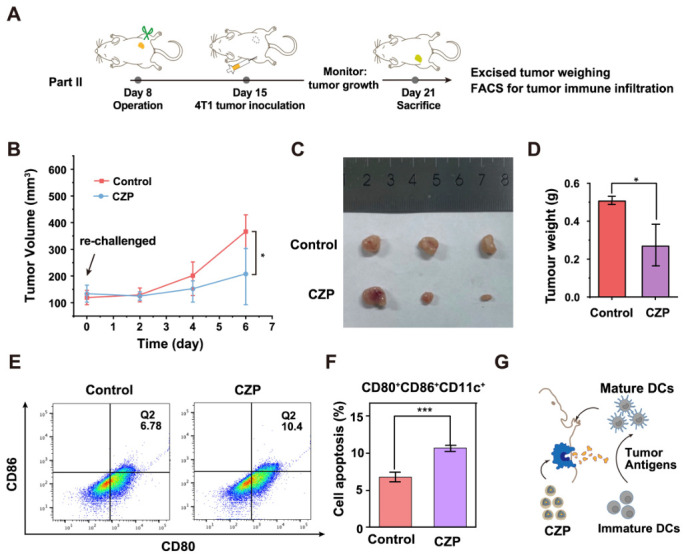
Re-challenge evaluation and tumor immune activation associated with CZP treatment. (**A**) Schematic illustration of the in vivo tumor re-challenge therapeutic protocol, detailing the timeline for primary tumor treatment, surgical excision, and subsequent secondary tumor inoculation. (**B**) Tumor growth curves of the secondary (distant) tumors after re-challenge in the PBS and CZP groups. (**C**,**D**) Macroscopic photographs of the excised secondary tumors at the end of the experiment (**C**) alongside the corresponding quantitative analysis of average tumor weights (**D**). (**E**,**F**) Flow cytometry analysis of mature tumor-infiltrating DCs (CD80^+^ CD86^+^ gated on CD11c^+^ cells) within the local tumor microenvironment in the PBS and CZP groups (**E**) and the corresponding quantitative statistical analysis (**F**). Pseudocolor indicates event density. (**G**) Schematic illustration of the proposed mechanism, in which CZP-induced tumor cell damage and immune activation may contribute to dendritic cell maturation and protection in the re-challenge setting. Data are presented as mean ± SD (*n* = 3). * *p* < 0.05, *** *p* < 0.001. For comparisons in (**D**,**F**), two-tailed Student’s *t*-test was used. For longitudinal data in (**B**), statistical analysis was performed using two-way repeated-measures ANOVA.

To evaluate whether prior CZP treatment was associated with measurable protection against secondary tumor inoculation, a tumor re-challenge model was established after primary treatment and tumor resection (Figure 6A). Primary 4T1 tumors on the right flank received nanotherapies. Subsequently, these treated tumors were surgically excised to simulate standard clinical resection. Following a short recovery phase, the mice were re-challenged with secondary 4T1 tumor cell inoculation on the distant left flank.

Following the secondary inoculation, the growth of the re-challenged tumors was monitored. Compared with the PBS control group, the CZP-treated group showed slower growth of secondary tumors (Figure 6B) and reduced tumor burden at the endpoint (Figure 6C,D). These findings suggest that prior CZP treatment was associated with protection in the re-challenge setting. Notably, these results should be interpreted as evidence of re-challenge protection associated with CZP treatment, rather than as a full comparative assessment of long-term immune memory across all formulations.

To further examine immune activation associated with CZP treatment in vivo, the local tumor immune microenvironment was investigated. Based on the in vitro findings of oxidative stress amplification, STING pathway activation, and dendritic cell maturation, we further analyzed the maturation status of intratumoral dendritic cells in the primary tumors. Flow cytometry showed that the CZP-treated group exhibited a higher percentage of mature intratumoral DCs (CD80^+^CD86^+^ gated on CD11c^+^ cells) than the PBS control group (Figure 6E,F). These results indicate that CZP treatment promoted dendritic cell maturation in vivo and may be associated with the observed protection in the re-challenge setting.

## 4. Discussion

Current cancer immunotherapy frequently struggles to eradicate immunosuppressive “cold” tumors [8,13]. To address this limitation, the ATP-responsive bimetallic CZP nanoplatform was developed. While conventional single-metal–organic frameworks and traditional chemodynamic agents have shown potential in tumor-targeted therapy [16,17,34], their overall efficacy is often constrained by insufficient catalytic activity within the tumor microenvironment [24,25,26]. In contrast, our CZP system utilizes a coordination-driven self-assembly strategy to enable ATP-responsive activation under tumor-associated conditions [35,36]. This responsive mechanism was designed to amplify oxidative stress within tumor cells while reducing nonspecific activation under physiological conditions.

Once inside the cells, CZP functions as a cascade catalytic platform. The PDA shell exhibits SOD-like activity. It converts natural oxygen compounds into H_2_O_2_. This process provides substrate support for subsequent Fenton-like reactions. Concurrently, released Cu-containing species participate in Fenton-like reactions. They utilize this generated fuel to produce •OH. In addition, CZP treatment was associated with intracellular GSH depletion, which may weaken the antioxidant defense of the tumor cells.

This chemical disruption was associated with a downstream biological response. The combination of oxidative stress amplification and Zn^2+^-associated mitochondrial damage may contribute to DNA leakage. This process was associated with activation of the cGAS-STING signaling pathway [27,28,29,30]. Molecular analysis showed increased phosphorylation of key proteins, including STING, TBK1, and IRF3. This molecular cascade was associated with increased dendritic cell maturation. These findings support enhanced antitumor immune activation, although they are not sufficient on their own to define a complete conversion of a “cold” tumor into a “hot” tumor.

The in vivo results further supported the antitumor potential of CZP. The CZP nanoplatform suppressed primary tumor growth and showed protection in the re-challenge model. Together with the observed dendritic cell maturation, these findings support the ability of CZP to promote antitumor immune activation. However, because the re-challenge experiment was performed as a follow-up evaluation of the primary formulation, the present results should not be interpreted as a full comparative analysis of long-term immune memory across all formulations. Additional studies will be required to further define the durability and breadth of the observed immune protection. A more comprehensive evaluation of tumor immune remodeling would require additional analysis of downstream effector immune populations, such as CD8^+^ T-cell infiltration and related functional markers.

## 5. Conclusions

In summary, this study demonstrates that the ATP-responsive bimetallic CZP nanoplatform can amplify oxidative stress, activate cGAS-STING-related immune signaling, and suppress tumor growth in a 4T1 cell-based breast cancer model. The observed protection in the re-challenge setting further supports its potential to promote antitumor immune activation. Future studies will be needed to further define the comparative durability and generalizability of this immune protection.

## Data Availability

The original contributions presented in the study are included in the article, further inquiries can be directed to the corresponding author.

## Supplementary Materials

The following supporting information can be downloaded at: https://www.mdpi.com/article/10.3390/jfb17040199/s1, Figure S1: Flow cytometry gating strategy and histopathological evaluation of major organs; Table S1: Quantitative ICP-MS determination of Cu content in the CZ sample.

## Abbreviations

The following abbreviations are used in this manuscript:

2-MIM	2-methylimidazole
ATP	Adenosine triphosphate
BMDCs	Bone marrow-derived dendritic cells
CCK-8	Cell Counting Kit-8
CDT	Chemodynamic therapy
cGAS	Cyclic GMP-AMP synthase
CZP	Cu/ZIF@PDA
DC	Dendritic cell
DLS	Dynamic light scattering
dsDNA	Double-stranded DNA
DTNB	5,5′-dithiobis-(2-nitrobenzoic acid)
EDS	Energy-dispersive X-ray spectroscopy
FTIR	Fourier transform infrared
GSH	Glutathione
ICB	Immune checkpoint blockade
ICD	Immunogenic cell death
MB	Methylene blue
MOF	Metal–organic framework
mtDNA	Mitochondrial DNA
PDA	Polydopamine
PI	Propidium iodide
ROS	Reactive oxygen species
SEM	Scanning electron microscopy
SOD	Superoxide dismutase
STING	Stimulator of interferon genes
TEM	Transmission electron microscopy
TME	Tumor microenvironment
WST	Water-soluble tetrazolium
XPS	X-ray photoelectron spectroscopy
XRD	X-ray diffraction
