# Supplementary material for: ATP-Responsive Bimetallic Metal–Organic Frameworks Amplify Oxidative Stress in the Tumor Microenvironment for Synergistic Chemo-Immunotherapy
Source: J Funct Biomater. 2026 Apr 19;17(4):199. doi: 10.3390/jfb17040199 (PMC13117820; doi:10.3390/jfb17040199)
Supplement: Supplementary file 1 [file jfb-17-00199-s001.zip › jfb-4230609-supplementary.pdf]

Supplementary Materials

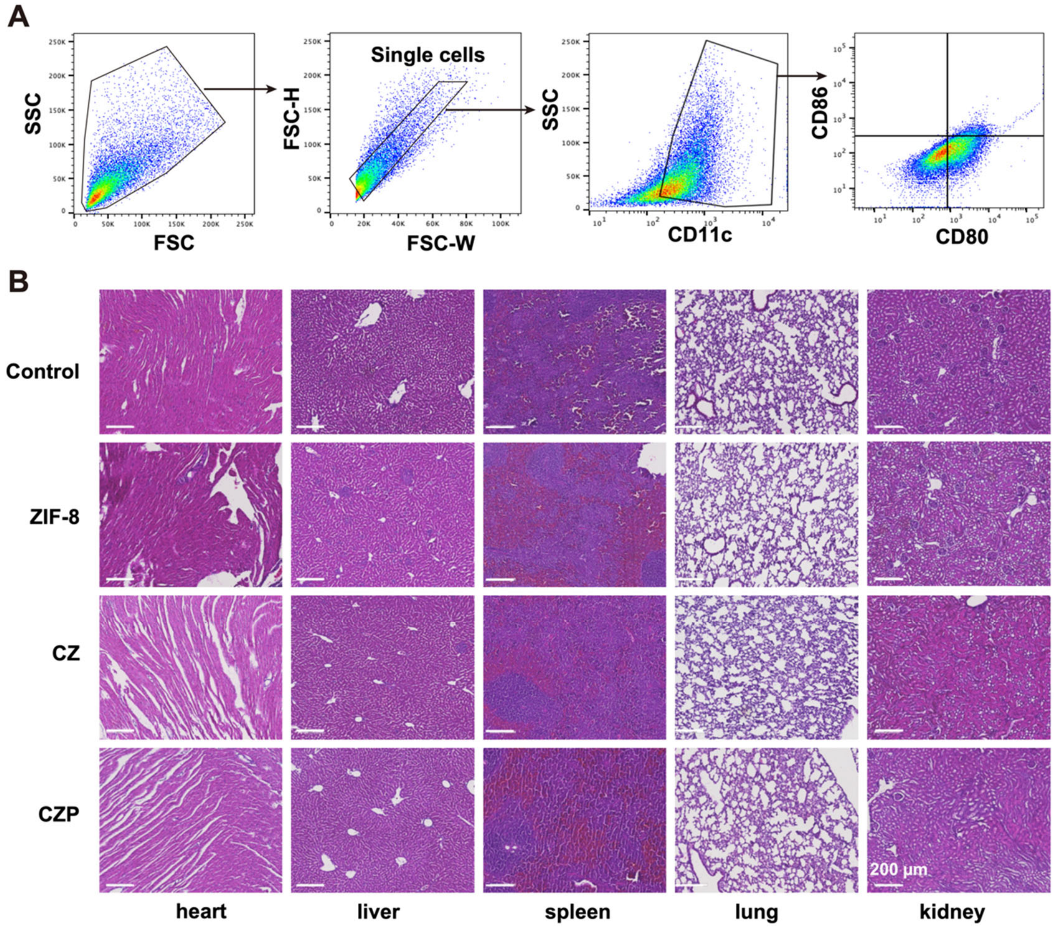

Figure S1. Flow cytometry gating strategy and histopathological evaluation of major organs. **(A)** The gating strategy used for the flow cytometry analysis of DCs. **(B)** Representative H&E staining images of major organs (heart, liver, spleen, lung, and kidney) collected from mice after different treatments, exhibiting no obvious histopathological abnormalities.

Table S1. Quantitative ICP-MS determination of Cu content in the CZ sample.

Sample	Sample mass (mg)	Cu concentration in digest (µg/L)	Cu content (µg/mg)	Cu content (wt%)
CZ	37.00	34308.28	34.31	3.43
